# Prevalence of hearing impairment in patients with mild cognitive impairment

**DOI:** 10.1590/S1980-57642008DN10300006

**Published:** 2007

**Authors:** Leonardo da Costa Lopes, Regina Miksian Magaldi, Mara Edwirges Rocha Gândara, Ana Carolina de Barros Reis, Wilson Jacob-Filho

**Affiliations:** 1,2,3,5 Geriatric Division of Hospital das Clínicas, Department of Internal Medicine, São Paulo University Medical School, São Paulo, SP, Brazil.; 4 Otorhinolaryngology Division of Hospital das Clínicas, São Paulo University Medical School, São Paulo, SP, Brazil.

**Keywords:** dementia, memory, hearing, audiometry, elderly, aged

## Abstract

The correlation between hearing and cognition is well established in dementia, but not in mild cognitive impairment (MCI).

**Objective:**

The aim of the present study was to define the prevalence of hearing impairment in elderly patients with MCI and in controls.

**Methods:**

Twenty-nine patients with MCI and 24 control subjects were analyzed. We evaluated memory and hearing impairments through clinical tests, including the Mini Mental Status Examination, Clinical Dementia Rating (CDR) and Hearing Handicap Inventory for the Elderly Screening (HHIE-S). Audiometries were performed in 22 patients with MCI and 19 subjects in a control group.

**Results:**

MCI patients showed more hearing complaints (68.9%) compared to the control group (25%) (p=0.001). No differences in the intensity of hearing complaints, measured by the HHIE-S, were detected. Nonetheless, differences between mean hearing threshold (MCI group=23.4±11.3dB and control group=16.0±10.1dB) (p=0.03) were identified.

**Conclusions:**

There is a significant association between MCI and hearing impairment. Hearing impairment in MCI patients may be a contributory factor to cognitive decline. This may however be related to the same neuropathological process, due to lesions of cortical areas related to hearing. The early diagnosis of hearing impairment in MCI patients may offer a more appropriate approach to this disease.

## Abstract (pt)

A relação entre audição e cognição está bem estabelecida em demências, porém não no comprometimento cognitivo leve (CCL).

**Objetivo:**

Propomos um estudo para determinar a prevalência de deficit auditivo em idosos portadores de CCL e controles.

**Métodos:**

Foram avaliados 29 pacientes com CCL e 24 controles. Analisamos as perdas de memória e de audição através de testes, como o Mini Exame do Estado Mental, o Escore Clínico de Demência e o HHIE-S (Hearing Handicap Inventory for the Elderly Screening). Vinte e dois pacientes com CCL e 19 controles foram submetidos a audiometrias.

**Resultados:**

O grupo CCL apresentou mais queixas auditivas (68,9%) se comparado com o controle (25%) (p=0.001). Não foram encontradas diferenças na intensidade da queixa auditiva, medida pelo HHIE-S. Foram detectadas diferenças entre a média dos limiares auditivos de pacientes com CCL (23,4±11,3 dB) e de controles (16,0±10,1 dB) (p=0,03).

**Conclusões:**

Existe significativa associação entre CCL e perdas auditivas. O deficit auditivo em pacientes com CCL pode ser um fator contribuinte para o declínio cognitivo ou estar relacionado a um mesmo processo neuropatológico, devido à lesão de áreas corticais relacionadas à audição. O diagnóstico precoce de perdas auditivas em pacientes com CCL pode permitir uma abordagem mais adequada desta doença.

The relationship between hearing impairment and cognitive decline has previously been demonstrated,^1^ showing that even mild or moderate hearing losses are correlated with poor performance in verbal memory.^2^. A link has also been established among hearing loss, depression and functional decline,^3^ especially evident in individuals with hearing threshold >35 dB.^4^

More than 90% of patients with Alzheimer disease (AD) have some kind of hearing loss.^5^ Although this relationship between cognition and hearing has been well studied in patients with dementia, the association in those with mild cognitive impairment (MCI) has not yet been assessed. MCI is a clinical entity, first described in the 1990s,^6^ and involves an intermediate state between normal cognitive aging and dementia.^7^ To date, no studies published in the English language have evaluated the prevalence of hearing impairment in such patients compared to normal control groups from a cognitive standpoint.

This paper aimed to analyze the prevalence of hearing impairment and its quantitative characteristics in elderly patients with MCI compared with normal elderly subjects from a control group, according to the cognitive standpoint – through a comparative transversal study.

## Methods

Twenty-nine patients with subjective memory complaints (MCI group) and 24 control-subjects without cognitive complaints (control group) were studied. Subjects were recruited from May to November of 2005. The MCI group was formed through assessment of subjects attending a memory clinic at the Geriatrics Department of Hospital das Clínicas – São Paulo University Medical School (SG-HC/FMUSP). The control group was formed through assessment of subjects followed by a multidisciplinary care group from SG-HC/FMUSP in 2003 and 2004. The study was approved by the Ethics Commission of the Institution. The inclusion criteria were:

(a) age of 60 years or over;
(b) Clinical Dementia Rating (CDR) of 0.5 in patients with cognitive complaints and CDR of 0 in controls;
(c) acceptance of an informed written consent.

The exclusion criteria were:

(a) presence of memory complaints among controls;
(b) presence of clinical signs of dementia, as well as scores below those expected for schooling level in the Mini Mental Status Examination (MMSE) as established in the following section;
(c) presence of any central nervous system disease, psychiatric diseases, such as anxiety and depression, severe inflammatory systemic diseases, as well as hypothyroidism, deficiency of vitamin B12, syphilis, renal and liver insufficiency;
(d) use of drugs that act on the central nervous system, mainly benzodiazepines, antidepressives and anticholinergic drugs;
(e) current or previous use of acetylcholinesterase inhibitor;
(f) current or previous use of hearing aids, or presence of visual and hearing losses severe enough to prevent clinical interview or proposed tests from being conducted.

All subjects were investigated for schooling level, comorbidities, medications being used, and hearing and memory complaints. Moreover, the following medical questionnaires were used: the MMSE,^8^, CDR,^9^, CAMCOG, a structured interview based on the cognitive section of Cambridge Examination for Mental Disorders of the Elderly^10^ and validated for the Brazilian population,^11^ Rivermead Behavioral Memory Test, which is divided into Rivermead 1 for scores of standardized profile and Rivermead 2 for scores of screening,^12^ Digit Span, which is divided into Digit Span 1 for direct-order numbers and Digit Span 2 for reverse-order numbers,^13^ Whispered Test^14^ and Hearing Handicap Inventory for the Elderly Screening – HHIE-S.^15^. Of the total subjects included in this study, 19 from the control group and 22 from the MCI group were submitted to an auditory examination, tonal audiometry and determination of speech reception threshold and percentage rate of speech recognition tests.

### Mini Mental State Examination

MMSE was assessed according to schooling level of the subject, using the criteria suggested for Brazilian population by Herrera et al.,^16^ considering a cut off point of <28 for subjects with more than 8 years at school, <24 for subjects who studied from 4 to 7 years, <23 for subjects who studied between 1 and 3 years, and <19 for illiterate subjects.

### Hearing tests

Subjects were investigated for hearing complaints and analyzed according to the HHIE-S, a scale that puts questions regarding the auditory performance in habitual situations. It is self applicable and assesses the degree of auditory loss, although is not capable of quantifying the level of the losses.^15^

All subjects were submitted to the Whispered Test and auditory examination. Twenty-two subjects from the MCI group and 19 from the control group were randomly selected and submitted to tonal audiometry. All tests were executed by the same examiner.

Tonal audiometry was performed by the MIDIMATE 622 audiometer, employing AZ 7 and Zodiac 901 middle-ear analyzers, studying air and bone conduction, in an acoustically isolated environment. The hearing threshold for frequencies from 250 to 8000 Hz was determined, as well as for frequencies of 500, 1000 and 2000 Hz, where means of these values for each ear were calculated.^17^ The result of the best ear was considered, in order to eliminate any confounding factor caused by unilateral hearing losses.^18^ Subjects who had a cerumen plug during otoscopy underwent removal before audiometry. Considering levels of severity of hearing loss, the threshold up to 25 dB is usually defined as normal, between 25 and 40 dB indicative of mild loss, between 40 and 65 dB as moderate loss, and over 65 dB as severe loss.^19^ We also calculated mean hearing thresholds at high frequencies (4000, 6000 and 8000 Hz), as well as the speech reception threshold and percentage rate of speech recognition.

### Statistical analysis

Both groups were compared regarding average age, schooling, comorbidities, medications used and scores on the tests applied. In addition, prevalence of hearing complaints and positivity of Whispered Test were compared as were averages of the hearing threshold obtained through audiometry. Continuous variables were compared using the Student t test while categorical variables were compared using the chi-square test, determining the p values. Proportions were compared using Fisher’s Exact Test. Binary logistic regressions were performed to assess risk ratios (RR). For calculations, the statistical program MINI TAB 14 was used.

## Results

Of 135 subjects initially considered, 53 were included in this study –29 of whom were part of the MCI group and 24 of the control group. The main causes of exclusion are shown in Table 1.

**Table 1:** Causes of exclusion of the patients and control subjects.

Causes	Patients (N=46)	Causes	Controls (N=36)
Depression	12	Depression	2
Dementia	8	Memory complaints	27
CDR=0	6	Language comprehension problems	2
Hypothyroidism	5	Hypothyroidism	1
B12 vitamin deficiency	3	B12 vitamin deficiency	1
Hearing aids	2	Hearing aids	2
Refusal	2	Refusal	1
Drugs with CNS effects	3		
Metastatic cancer	1		
Anxiety	1		
Severe hearing impairment	1		
Severe visual impairment	1		
Severe CNS disease	1		

CDR, clinical dementia rating; CNS, central nervous system.

Table 2 shows clinical features of subjects and the result of tests applied.

**Table 2:** Characteristics of groups.

Characteristic	Control group	MCI group	p
Number of patients	24	29	----
Age (y)	70.3±5.4	75.0±5.6	0.004
Female (%)	75	79.3	0.709
Number of comorbidities	4.1±1.7	3.0±1.4	0.014
Number of drugs	4.4±1.7	3.5±2.3	0.113
Schooling (y)	5.7±3.5	5.4±5.5	0.787
MMSE*	26.5±2.8	25.9±2.9	0.413
CAMCOG^†^	92.5±8.4	81.4±10.1	0.000
Memory subscale^‡^	22.0±2.5	19.9±3.7	0.015
Rivermead 1^§^	19.2±2.4	15.5±3.8	0.000
Rivermead 2^||^	8.6±1.6	6.2±2.0	0.000
Digit Span 1 (attention/concentration)^¶^	5.7±1.7	4.9±1.2	0.061
Digit Span 2 (operational memory)**	4.5±1.9	3.8±1.2	0.172
Hearing Complaints (%)	25	68.9	0.001
Altered Whispered Test (%)	12.5	41.3	0.017
HHIE-S^††^	18.7±11.6 (n=6)	16.1±10.5 (n=20)	0.643

* Mini Mental State Examination. Score: 0-30. Lower values represent higher deficits;

† Cambridge Examination for Mental Disorders. Score: 0-107. Lower values represent higher deficits. Roth12 describes a cut-off of 79-80 for demented patients;

‡ CAMCOG´s subscale related to memory. Score: 0-27. Lower values represent higher deficits;

§ Rivermead's Test (score of standardized profile). Score: 0-24. Lower values represent higher deficits. (greater than or equal to 22: normal; 17 to 21: mild deficit; 10 to 16: moderate deficit; lower than 10: severe deficit);

|| Rivermead's Test (selection score). Score: 0-12. Lower values represent higher deficits (greater than or equal to 1: normal; 7 to 9: mild deficit; 3 to 6: moderate deficit; lower than 3: severe deficit);

¶ Direct order. Score: 0-14. Lower values represent higher deficits;

** Reverse order. Score: 0-14. Lower values represent higher deficits;

†† Hearing Handicap Inventory for the Elderly Screening. Score: 0-40. Higher values represent higher deficits; MCI, mild cognitive impairment.

The MCI group was older (75.0±5.6 years of age) than the control group (70.3±5.4 years of age), p<0.05. In addition, a greater number of comorbidities was observed in the control group (4.1±1.7) compared to the MCI group (3.0±1.4), p<0.05. The most common diagnoses were similar for both groups: systemic arterial hypertension (54.1% and 58.6%), dyslipidemia (50% and 51.7%), and osteoarticular diseases (45.8% and 41.3%) respectively. The most common drugs used by both groups were also similar: calcium carbonate (50% and 44.8%), statins (41.6% and 44.8%) and angiotensin-converting enzyme inhibitors (41.6% and 37.9%) respectively.

Regarding other clinical features, no statistical differences between the groups were observed, nor in relation to the score on the MMSE and Digit Span.

However, scores of the two groups were different on the CAMCOG (92.5±8.4and 81.4±10.1for control and MCI group, respectively p<0.05) and in their subscale of memory (22.0±2.5and 19.9±3.7, respectively, p<0.05), as were scores on the Rivermead Behavioral Test (Rivermead 1: 19.2±2.4and 15.5±3.8; Rivermead 2: 8.6±1.6 and 6.2± 2.0, respectively, p<0.05).

The MCI group presented more hearing complaints (25% and 68.9%, control and MCI group, respectively, p<0.05). The risk ratio (RR) for patients with MCI presenting hearing complaints was 6.6. No significant differences in proportions of normal otoscopies were detected between the two groups. The Whispered Test was abnormal in 12.5% of control group subjects and 41.3% of the MCI group (p<0.05). The risk ratio (RR) of a patient with MCI being positive on the Whispered Test was 4.9. No differences in the severity of hearing complaints were detected, according to the HHIE-S. The mean hearing threshold of the best ear assessed was statistically different between the two groups (16.0±10.1dB and 23.4±11.3dB, control and MCI group, respectively). Nevertheless, these thresholds lie within the range of audiometric normality. When we analyzed hearing means, classifying them individually as normal (up to 25 dB) or abnormal (>25dB), we observed that 10.5% of the control group subjects showed abnormal thresholds, while this was the case in 31.8% of the MCI group (p=0.09).

The analysis of hearing thresholds at high frequencies (4-8 KHz) revealed differences between control (33.2±15.4dB) and MCI groups (46.2±20.1dB), p<0.05. No significant differences between speech reception threshold and percentage rates of speech recognition were detected. Table 3 shows the results of audiometric tests.

**Table 3:** Audiometric tests.

	Control group (n=19)	MCI group (n=22)	p
Hearing Threshold (dB, 0.5-4 KHz)	16.0±10.1	23.4±11.3	0.033
Hearing Threshold >25 dB (%)	10.5	31.8	0.092
Threshold - High Frequencies (in dB, 4-8 KHz)	33.2±15.4	46.2±20.1	0.024

MCI, mild cognitive impairment.

## Discussion

Some papers have used a CDR score of 0.5 as a criterion for MCI, formally known as “mild dementia status”.^6^ Nonetheless, other authors have determined differences between subjects with CDR of 0.5 and with MCI, particularly if the criteria defined by Petersen are considered.^20^ However, these were reviewed recently by the European Alzheimer disease Consortium.^21^ Thus, diagnostic elements for MCI were initially defined as the presence of memory complaints reported by the patient and/or relatives, with decline of cognitive function in the last year, cognitive alterations in a medical assessment, along with impairment of memory or other cognitive domains, without effect on daily life, and in the absence of dementia. According to these criteria, specific tests for assessment of cognitive losses were not devised, nor were performance deficits that classify the disease. Given the absence of neuropsychological assessments in this paper, the criterion of CDR of 0.5 was chosen. All patients diagnosised as MCI cases were of the mnestic type.

The fact that the MCI group was more aged than the control group may be explained by the fact that these patients at greater risk of developing dementia syndromes, where age represents a risk factor. As this study was not conducted following the case-control standard-paired according to age, we tried to detect markers in the sample of our population in order to ensure equality between the studied groups so as to facilitate comparison. Since the main objective of this study was related to hearing complaints, most likely intensified by the aging process, we also investigated the frequency of hearing complaints in the medical literature. Among normal subjects, 33% of them aged between 64 and 74 years, and 45% of those aged between 75 and 84 years, tend to present hearing impairment,^22^ suggesting that the rate of 68.9% of hearing complaints in patients with MCI found in this study, was not a consequence of age but associated with cognitive impairment. In the general elderly population, the prevalence of hearing impairment varies from 30-60% and may reach 79% in the demented.^23^

The only variable different between groups was the number of comorbidities. Despite their advanced age, patients with MCI presented a lower number of diseases compared to the control group. This data emphasizes the fact that hearing impairment seen in this group was not associated with worse general clinical condition.

In assessment with CAMCOG, we observed that even though none of the groups had scores that suggested dementia features, values differed from each other. Similarly, when Bottino^24^ analyzed 41 subjects, controls were detected with normal memory and MMSE scores >28, CAMCOG mean of 91±2 whereas patients with MCI scored a mean of 82±4 (mean age of 73.05 years and schooling level of 5.61 years) – in line with that seen in our sample.

On the memory subscale of CAMCOG, scores also differed, showing loss of memory in patients with MCI. In the Rivermead Behavioral Test, the MCI group obtained lower scores with a statistically significant difference.

Alterations of memory observed were not a consequence of attention disorders, as suggested by the Digit Span 1 score (direct-order). The absence of differences in reverse-order of repetition of digits (Digit Span 2) suggested that operational memory (very short term) was similar in both groups. Furthermore, long-term memory is expected to be compromised in the MCI group, justifying the worse performance in cognitive tests.

In spite of the higher frequency of hearing complaints in patients with MCI, the severity of complaints was not different between both groups, demonstrated by scores on the HHIE-S. Vesterager^25^ demonstrated that the Handicap does not correlate correctly with scores of self-perception of hearing losses. Nonetheless, there is the possibility that patients with MCI have a certain loss of critical ability in relation to their own hearing deficiency.

There was difference between mean hearing thresholds obtained through audiometry between the frequencies of 500 and 2000 Hz, often used to define the level of hypoacusis. However, the thresholds observed in both groups remained within the range of normality. Speech reception threshold and percentage rate of speech recognition were also similar, suggesting that hearing complaints presented by the subjects cannot be accounted for by the differences detected in frequencies from 500-2000 Hz. Among the elderly, neurosensory losses are common in 90% of cases,^17^ associated with presbyacusis. In presbyacusis there is bilateral and gradual loss of hearing sensitivity for high-frequency sounds^26^, mainly in noisy environments. In the analysis of high frequencies (4-8 KHz), we observed differences between the groups, with mild loss in the control group and moderate in the MCI group, suggesting that MCI patients have more severe presbyacusis, which may be caused by a cognitive worsening since this contributes to poor sound comprehension, but may also be a consequence of the neurodegenerative process related to memory losses. Another possibility is that this phenomenon is an indicator of severity of the process of cognitive loss and that hearing complaints of patients with MCI are related to alterations in central auditory processing. Central auditory dysfunction was evident in studies on patients with mild AD, compared to controls of the same age. The peripheral auditory system however, seems to be similar in normal and demented patients.^27^

Presbyacusis may be accompanied by degeneration of central auditory structures and auditory cortex,^28^ formerly called “central presbyacusis”.^29^ Hearing loss among patients with dementia is not limited to peripheral alterations, but also involves reduction in speed of auditory processing.^30^ Thus, hearing losses may not be properly characterized by audiometric analysis, where assessment of the central auditory function is also required.^28^ Patients with AD show evidence of degeneration of structures related to auditory processing, including the colliculus, medial temporal lobes, and auditory cortex, where neuritic plaques and neurofibrillar tangles have been detected in these areas.^31^ Furthermore, here is histopathological evidence of impairment of the medial geniculate body and inferior colliculus in patients with AD.^32^ Moreover, prefrontal cortex is affected more precociously by amyloid-beta plaques in animal models,^33^ which may reduce the response of auditory cortical neurons given the direct connections between these two areas.^34^ Prefrontal cortex lesions are associated with an increase in amplitude of evoked potentials generated in the auditory cortex.^35^

Central auditory dysfunction has previously been characterized through responses of brainstem in patients with mild AD, who did not show higher rates of peripheral hearing impairment compared to controls.^17^ Pekkonen^36^ investigated how auditory processing is correlated with damage in superior cortical functions through the use magnetoencephalography techniques. Besides, he observed that inter-hemispheric auditory processing in AD is slower on the same side of the brain stimulated by sounds. In addition, he observed neurodegenerative alterations in primary auditory cortex, as well as in thalamus and inferior colliculus.^37^ When Golob^38^ assessed motor reaction time after an auditory stimulus, he detected alterations in modulation of auditory cortex of subjects with MCI resulting from neuropathological alterations in associative cortical areas.

However, in another study, subjects with CDR of 0.5 showed signs of central, and not peripheral, hearing deficiency.^17^ Studies employing positron emission tomography (PET) have showed reduction of metabolism of glucose in the temporo-parietal region of patients with dementia, including the auditory cortex.^39^

The most important limitation of this study is the absence of grouping for age, which affects the conclusions. Future studies should evaluate larger groups, paired according to age, while addressing peripheral and central auditory losses.

Early diagnosis of patients with MCI and the study of its epidemiological characteristics may allow an earlier clinical approach, particularly when risk factors are closely associated to the development of dementia, as is the case of hearing impairment.
